# Molecular profile of non-small cell lung cancer in a predominantly Black African population in Kenya: A single institution review

**DOI:** 10.1016/j.tranon.2025.102348

**Published:** 2025-03-19

**Authors:** Sitna Mwanzi, Shahin Sayed, Swati Das, Jonathan Wawire, Priscilla Njenga, Jasmit Shah, Asim Jamal Shaikh

**Affiliations:** a Department of Hematology-Oncology, Aga Khan University Hospital, Nairobi, Kenya; b Department of Pathology, Aga Khan University Hospital, Nairobi, Kenya; c Department of Internal Medicine, Aga Khan University, Hospital, Nairobi, Kenya; d Brain and Mind Institute and Department of internal Medicine, Aga Khan University Hospital, Nairobi, Kenya; e Sultan Qaboos Comprehensive Cancer Care and Research Centre (SQCCCRC) Muscat, Oman

**Keywords:** NSCLC, Driver mutations, Precision medicine

## Abstract

**Introduction:**

Targeted therapies for patients with non-small cell lung cancer (NSCLC) have significantly improved the outcomes for patients with oncogenic driver mutations. Testing for epidermal growth factor receptor (EGFR) mutations, anaplastic lymphoma kinase (ALK) rearrangement, c-ros oncogene 1 (ROS1) rearrangement and programmed death ligand 1 (PDL 1) testing are now highly recommended for all patients with NSCLC. There are clear geographical differences in the rate of driver mutations with higher EGFR mutation rates in the Asian population in comparison to the Caucasian population. Little is known about the rate of oncogenic driver mutations in predominantly Black African populations like those living in the East African region. This study describes the oncogenic driver mutations found in lung cancer patients diagnosed in a single institution in Kenya.

**Patients and methods:**

We retrospectively reviewed the charts of patients who had a pathological diagnosis of NSCLC at Aga Khan University Hospital, Nairobi (AKUHN) between January 2012 and December 2022. Data was analyzed for socio-demographic characteristics, smoking status, clinical stage, histological sub-type and presence or absence of driver mutations.

**Results:**

123 cases of non-small cell lung cancer (NSCLC) were included in the analysis. The median age at diagnosis was 62 years (IQR: 53.0 – 71.0) and 41.5 % (*n* = 51) of patients were under 60 years at time of diagnosis, 47.2 % (*n* = 58) were female and 78.9 % (*n* = 97) were of Black African descent. Only 29.4 % (*n* = 30) were known smokers whereas 73.2 % (*n* = 90) had stage IV disease. Adenocarcinoma was the most common sub-type in 85.4 % (*n* = 105) patients and 60 % of cases had EGFR testing done and a mutation was detected in 35 % (*n* = 26/74) patients tested. ALK and ROS rearrangement was positive in 19.5 % (*n* = 8/41) and 6.0 % (*n* = 2/33) patients tested respectively. Only 23.5 % (*n* = 8/34) of patients tested for PDL1 had an expression of >1 %. There was no significant association between gender, ethnicity and smoking with EGFR mutation.

**Conclusion:**

In our setting, EGFR testing was the most common molecular test done for lung cancer with a positive rate like that reported in Asian communities. Further studies are needed in a larger population to define further the molecular profile of lung cancer in Sub Saharan Africa. Access to testing will enhance targeted therapies for patients leading to improved outcomes.

## Highlights

• Geographical differences in incidence, mortality and prevalence of driver mutations exist.
• Very little is known on the prevalence of different driver mutations in predominantly Black population in Eastern Africa.
• Our study highlights similar EGFR mutation positive rates in Black Africans similar to that seen in Asian population.
• There was no difference in EGFR mutation status with regards to gender, ethnicity or smoking status.

## Introduction

Lung cancer continues to be the most diagnosed cancer globally and is the most common cause of cancer related mortality. In 2022, lung cancer accounted for 1.8 million deaths globally. Geographical differences in incidence and mortality exist with highest being in Micronesia, North America, Europe, Australia, and the lowest in East, Central and West Africa. In Kenya, lung cancer is the 11th most diagnosed cancer and cause of cancer deaths with 903 new cases and 822 deaths in 2022 [1].

From as early as 2009, targeted therapy in non-small cell cancer has been shown to be beneficial in patients with select mutations in EGFR [2] ushering the era of precision medicine in NSCLC. It is well described that different population groups have different rates of EGFR mutation with higher prevalence of EGFR mutations in the Asian population, reported to be in the range of 30- 60 % in contrast to the rates for Caucasian population reported to be around 10–15 % [[3], [4], [5]].

Other important driver mutations such as anaplastic lymphoma kinase (ALK) rearrangement has a reported prevalence of 2- 7 % [6] and rearrangements of the ROS1 gene occurring in 1–2 % of non-small cell lung cancers (NSCLCs) [7] . In addition to the molecular alterations, tumor expression of the immune checkpoint molecule, programmed death ligand (PD-L) has important therapeutic implications for patients with lung cancer [8].

Management of lung cancer in resource limited setting in the era of targeted therapy and immunotherapy presents new challenges and opportunities for clinicians and patients. Whereas there is hope for better outcomes, it comes with increased burden of testing, which is limited by lack of testing capability locally, cost of testing and delayed results. Even when testing is done, targeted therapies and immunotherapy may not be available or affordable to most patients [9].

Because of these factors, there is paucity of data on the molecular profile of NSCLC in Black African patients specifically those from Sub-Saharan Africa. The available studies have small number of patients tested for molecular profile. Legius et al. reported on a case series of 6 patients of Black African descent with 3 of 6 and 1 of 6 having EGFR mutation and ALK rearrangement respectively [10]. A study from Ghana had only 4 of 90 patients with lung cancer tested for EGFR mutation and 2 of those had an EGFR mutation [11]. Finally, Manikariza et al. reported on 12 patients who underwent molecular testing in Rwanda with EGFR mutation being the most commonly identified mutation in 33 % of patients [12].

Our study aims to increase the knowledge on molecular profile of Black African patients with NSCLC by describing the clinicopathological characteristics and molecular profile of patients diagnosed with non-small cell lung cancer at a tertiary hospital in Kenya.

## Patients and methods

### Patients

All patients with primary NSCLC who were diagnosed and/or treated at the AKUHN between January 2012 and December 2022 were included in this retrospective study. Patient charts were reviewed for sociodemographic data including age at diagnosis, gender, ethnicity, smoking status, and stage at presentation. Pathology and molecular data included the histologic subtype of lung cancer and where available the results of additional molecular testing specifically EGFR, ALK, ROS and PDL1 expression.

### Ethical approval

The study protocol was approved by the Aga Khan University Institutional Research and Ethics Committee (Ref 2019/REC-10 (v1)). All the data were collected and de-identified by SM and the research nurse.

### Outcome variables

Outcome variables included clinical stage, histological subtype and presence/absence of driver mutations.

### Statistical analysis

Descriptive statistics was used where medians and interquartile ranges (IQR) was used for continuous data and frequencies and percentages for categorical data to summarize patient clinical characteristics, histological features and data on molecular profile. Data was abstracted into a spreadsheet after which analysis was done on SPSS® version 23 (IBM corporation, Armonk, New York, USA).

## Results

### Patient characteristics

Between January 2012 and December 2022, 123 patients with non-small cell lung cancer were identified with an average of 11 cases per year. The median age at diagnosis was 62.0 years (IQR: 53.0–71.0). Age distribution showed that 41.5 % (*n* = 51) were between 30 and 59 years, 30 % (*n* = 37) were between 60 and 69 years and 28.5 % (*n* = 35) were over the age of 70 years. Among the patients, 47.2 % (*n* = 58) were females. The majority were of Black African descent at 78.9 % (*n* = 97), while 10.6 % (*n* = 13) patients were of Asian and Caucasian descent respectively. Smoking status was recorded for most patients, with 29.4 % (*n* = 30) identified as smokers and 70.6 % (*n* = 72) as non-smokers. About 73.2 % (*n* = 90) of patients were diagnosed with stage IV disease with stage unknown for 15.4 % (*n* = 19) of patients. Two patients diagnosed with earlier stages of disease and treated in curative setting, developed disease recurrence during the period of follow up (Table 1).

**Table 1:** Sociodemographic Characteristics of NSCLC patients.

(N = 123)	n	%
Age (years) (median [IQR]	62.0 [53.0, 71.0]	
Age at diagnosis (years)		
30–49	22	17.9
50–59	29	23.6
60–69	37	30.1
70–79	28	22.8
80 and above	7	5.7
Gender		
Male	65	52.8
Female	58	47.2
		
Ethniciity		
African	97	78.9
Asian	13	10.6
Caucasian	13	10.6
Smoking status		
Smoker	30	24.4
Non smoker	72	58.5
Unknown	21	17.1
Stage at diagnosis		
I	6	4.9
II	2	1.6
III	6	4.9
IV	90	73.2
Unknown	19	15.4

Pathology information was available for all 123 patients. Adenocarcinoma was the most common histological type identified in 85.4 % (*n* = 105) of the patients followed by squamous cell carcinoma in 11.4 % (*n* = 14) of the patients. Additionally, two patients had a histological diagnosis of large cell neuroendocrine carcinoma and one each with adeno-squamous carcinoma and NSCLC not otherwise specified (NSCLC—NOS).

The most common driver mutation tested was EGFR mutation performed in 60 % (*n* = 74) patients. An EGFR mutation was identified in 35 % (*n* = 26/74) of the patients with deletion of exon 19 mutation being the most common identified in 10 of the 26 patients followed by exon 21 mutation in 5 patients and the rest with exon 18 and 20 (Table 2).

**Table 2:** Driver Mutations in NSCLC.

	EGFR Mutation	ALK rearrangement	ROS Rearrangement	PDL 1 Expression
Performed (n/%)	74 (60.2 %)	41 (33.3 %)	33 (26.8 %)	34 (27.6 %)
Not Performed	49 (39.8 %)	82 (66.7 %)	90 (73.2 %)	89 (72.4 %)
Detected/Present	26 (35.1 %)	8 (19.5 %)	2 (6.0 %)	8 (23.5 %)
Not detected/Absent	48 (64.9 %)	33 (80.5 %)	31 (94.0 %)	26 (76.5 %)

Of the 26 mutations, 57.7 % (*n* = 15) mutations were detected in males and 42.3 % (*n* = 11) in females. From the Black African tested (*n* = 57), 38.6 % were positive whereas 33.3 % of the Asian were positive and 12 % of the Caucasian were positive. There was no statistically significant difference in EGFR mutation based on gender, ethnicity or cigarette smoking (Table 3, Table 4).

**Table 3:** Ethnicity and EGFR mutations in NSCLC.

Ethnicity/EGFR Mutation	Tested	Detected (n/% of those tested)
Black African	57	22 (38.5)
Asian	9	3 (33.3)
Caucasian	8	1 (12.5)
Total	74/123	26/74

**Table 4:** Gender, Ethnicity and Smoking Status in relation to EGFR mutations.

		EGFR		P value
		Detected	Not Detected	
Gender	Male	15 (57.7 %)	24 (50.0 %)	0.628
	Female	11 (42.3 %)	24 (50.0 %)	
Ethnicity	Black	22 (84.6 %)	35 (72.9 %)	0.443
	Asian	3 (11.5 %)	6 (12.5 %)	
	Caucasian	1 (3.8 %)	7 (14.6 %)	
Smoking	Yes	20 (87.0 %)	29 (70.7 %)	0.220
	No	3 (13.0 %)	12 (29.3 %)	

ALK rearrangement was positive is 8 (19.5 %) of 41 patients tested while 2 (6 %) of 33 patients tested for ROS rearrangement had a positive test. 8 (23.5 %) of 34 patients tested for PD-L1 had an expression of >1 % with the highest being 65–70 %.

Treatment and outcome data was missing for >50 % of patients and was therefore not included in the analysis.

## Discussion

Although lung cancer is the most common cancer and the leading cause of mortality worldwide, the cases reported from sub -Saharan Africa and specifically East Africa are very low [1,2]. We diagnose and treat an average of 11 NSCLC cases per year which is similar to that previously reported from Western Kenya [13]. The low incidence reported could be multifactorial. In a setting endemic for tuberculosis, it is highly likely that a number of patients are misdiagnosed as having tuberculosis [14]. There are also challenges in accessing diagnostic services such as radiology, bronchoscopy with image guided biopsy and comprehensive pathology.

Our case series shows that most of our patients were over the age of 50, of Black African descent, male and nonsmokers. The median age of diagnosis and age range distribution is our study is similar to studies in other regions of Africa and Asia. The median age in Tunisia and India was 64 and 62 years respectively and the majority of patients were older than 50 years of age similar to our study [15,16]. The gender distribution is similar to South Africa with almost two-thirds of patients being of male gender [17]. This is in contrast to Tunisia where Missaoui et al. report a higher proportion of male patients in their series where 94.6 % of their patients were male reflecting the prevalent practice of water pipe smoking in the Middle Eastern North African (MENA) region which is more common in males than females [15].

Close to a third (29 %) of our patients with known smoking history were smokers. This rate is higher than the smoking rate in the general population in Kenya which is estimated at between 13.5 and 17.1 % [18,19]. It is well documented that smoking increases the risk of lung cancer significantly and accounts for 80–90 % of all lung cancer cases [20].

Majority of our patients presented with advanced stage IV disease with rates similar to those seen in Tunisia (79.9 %), and India (67%) but higher than the United Kingdom (43 %) [[15], [21], [22]]. Adenocarcinoma was the most common histological subtype occurring in 85.4 % of our patients followed by squamous cell carcinoma. This rate is higher than the reported literature reported rate from rest of the world of about 40–60 % for adenocarcinoma reflecting the changing histological landscape seen globally with reversal and reduction in squamous cell carcinoma [3].

EGFR mutation was the most common molecular signature tested for and detected. This is not unexpected as this was the first biomarker that was recommended for routine testing in guidelines as far back as 2013. Our testing rate of 60 % is similar to other countries. There is wide variability in EGFR testing globally ranging from 38 % in Brazil to 91 % in Taiwan [23]. The rate of testing is likely to increase over time as the capability for testing has been introduced locally as of 2023 at access pricing with shorter turnaround time.

The rate of EGFR mutation of 35 % is closer to the rate for the Asian population of 30–50 % [5]. We had a low number of patients of Asian origin in our study who had a similar rate of testing to the patients of African descent. The higher positive rate in male patients possibly reflects the distribution of patients included in the study. Our EGFR positive rate is higher than that reported in South Africa of 22 % by Chan et al. In their study, majority of patients were Caucasian and female whereas we had a higher Black population with more males. 23 % of the 31 Black African patients in their study had a positive rate while our positive rate was 38 % for Black African patients. Similar to the South African study, the positivity rate was higher in non-smokers [24].

Our prevalence of EGFR mutation was higher than that reported in the North Africa which ranges from 17.5 to 21.5 %. This is likely due to a higher rate of smoking in North Africa making EGFR mutations less likely despite similar rates of adenocarcinoma [25]. It is also higher than that reported in Black Americans reported at 14 % in a study by Cheng et al. [26].

Likely due to the low numbers tested, we did not find any statistical significance in EGFR mutation rate based on gender, ethnicity and smoking status. It is well described that EGFR mutation rate is higher in females, those of Asian descent and non-smokers [27]. Further work is needed with larger number patients in order to clarify any associations.

The number of patients tested for ALK rearrangement, ROS rearrangement and PD 1 expression was relatively low in our setting mainly due to high cost of the tests and unavailability of the test locally. In addition, treatments directed to these rearrangements and immunotherapy are generally more expensive than EGFR directed therapies. Our ALK testing rate of 33 % is considerably lower than in other settings [24]. The low numbers tested make it difficult to make inferences on the data. For example, our ALK positive rate of 24 % is much higher than what is reported in literature 2–7 % [6].

There were several limitations in our study. As this was a retrospective study, some clinical information was missing particularly the treatment and outcome data of patients. This would have been important to show outcome based on targeted therapy. Since our lab acts a referral resource for the region, many of the patients diagnosed at our laboratory were getting treatment elsewhere or lost to follow up. Partial testing was also done in most patients as the full molecular profile as recommended in guidelines is beyond the reach of most patients.

## Conclusion

EGFR mutation testing is the most common molecular test done for non-small cell lung cancer in our setting with a positive rate similar to that seen in the Asian population. This information is useful for clinicians and policy makers to advocate for locally available testing at reasonable pricing with improving access to targeted therapy which have revolutionalised outcomes for lung cancer patients globally.

## CRediT authorship contribution statement

**Sitna Mwanzi:** Writing – review & editing, Writing – original draft, Visualization, Project administration, Methodology, Investigation, Formal analysis, Data curation, Conceptualization. **Shahin Sayed:** Writing – review & editing, Methodology, Investigation, Conceptualization. **Swati Das:** Writing – review & editing, Investigation, Conceptualization. **Jonathan Wawire:** Writing – review & editing, Investigation, Data curation. **Priscilla Njenga:** Investigation, Data curation. **Jasmit Shah:** Validation, Formal analysis. **Asim Jamal Shaikh:** Writing – review & editing, Visualization, Supervision, Methodology, Conceptualization.

## Declaration of competing interest

The authors declare the following financial interests/personal relationships which may be considered as potential competing interests: Honoraria from speaking engagement. Sitna Mwanzi – Astra Zeneca, MSD, Beacon, Janssen (<2500USD in last 12 months). Shaheen Sayed – Astra Zeneca (<2500USD in last 12 months). Conference travel support (ESMO,ASCO). Sitna Mwanzi – Janssen, MSD, Beacon in the last 24 months
